# Functional Cure for Hepatitis B Virus: Challenges and Achievements

**DOI:** 10.3390/ijms26083633

**Published:** 2025-04-11

**Authors:** Oren Shechter, Daniel G. Sausen, Harel Dahari, Andrew Vaillant, Scott J. Cotler, Ronen Borenstein

**Affiliations:** 1 Eastern Virginia Medical School, Norfolk, VA 23501, USA; shechto@odu.edu; 2 Naval Medical Center Portsmouth, Portsmouth, VA 23708, USA; daniel.g.sausen.mil@health.mil; 3 The Program for Experimental and Theoretical Modeling, Division of Hepatology, Department of Medicine, Stritch School of Medicine, Loyola University Chicago, Maywood, IL 60153, USA; hdahari@luc.edu (H.D.); scotler@luc.edu (S.J.C.); 4 Replicor Inc., 6100 Royalmount Ave., Montreal, QC H4P 2R2, Canada; availlant@replicor.com

**Keywords:** hepatitis B virus (HBV), functional cure, HBsAg, PEG-IFNα therapy, nucleos(t)ide analog (NA) therapy, siRNA therapy, therapeutic vaccine, immunological markers

## Abstract

The Hepatitis B Virus (HBV) presents a formidable global health challenge, impacting hundreds of millions worldwide and imposing a considerable burden on healthcare systems. The elusive nature of the virus, with its ability to establish chronic infection and evade immune detection, and the absence of curative agents have prompted efforts to develop novel therapeutic approaches beyond current antiviral treatments. This review addresses the challenging concept of a functional cure for HBV, a state characterized by the suppression of HBV and HBsAg, mitigating disease progression and transmission without a complete cure. We provide an overview of HBV epidemiology and its clinical impact, followed by an exploration of the current treatment landscape and its limitations. The immunological basis of a functional cure is then discussed, exploring the intricate interplay between the virus and the host immune response. Emerging therapeutic approaches, such as RNA interference-based interventions, entry inhibitors, nucleic acid polymers, and therapeutic vaccines, are discussed with regard to their success in achieving a functional cure. Lastly, the review underscores the urgent need for innovative strategies to achieve a functional cure for HBV.

## 1. Introduction

Hepatitis B virus (HBV) is a partially double-stranded DNA member of the *Hepadnaviridae* family [1]. Infection with HBV can present with acute or chronic hepatitis and can progress to cirrhosis and hepatocellular carcinoma (HCC) [2]. Hepatitis B is known to pose a significant burden on global health. As of 2022, the World Health Organization (WHO) estimated that there were 254 million people living with chronic hepatitis B (CHB), with 1.2 million new infections as well as 1.1 million deaths annually globally [3]. The burden of disease is particularly pronounced in the African and Western Pacific regions, where perinatal and early childhood transmissions remain the predominant modes of infection. Despite the availability of an effective vaccine, the global coverage of the timely birth dose remains suboptimal, reaching only 45% worldwide and as low as 18% in Africa [3]. This reflects the persistent challenge in preventing vertical and early-life transmissions, despite the birth-dose vaccine being 90–95% effective when given within 24 h of birth [4].

Treatment advances over the past several decades have resulted in improved quality of life and life expectancy for persons living with CHB. Pegylated interferon-α (PEG-IFNα) and nucleos(t)ide analogs (NAs) can suppress HBV DNA, resulting in decreased liver inflammation and fibrosis, as well as decreased rates of cirrhosis and HCC [2]. However, such treatments have failed to result in a complete (sterilizing) cure for patients living with CHB, which is defined as the elimination of all genetic traces of HBV infection in the liver, including covalently closed circular HBV DNA (cccDNA) and chromosomally integrated HBV DNA.

Hepatitis B cccDNA is a stable minichromosome that is found in the nucleus of infected hepatocytes, serving as the transcriptional template for HBV replication. It is highly resistant to antivirals, and no effective mechanisms of elimination have been discovered [5]. cccDNA serves as a template for transcription and represents a key viral product in maintaining chronic infection [6,7]. Therefore, the eradication of HBV infection would require the clearance of cccDNA. Because a liver biopsy is needed to assess cccDNA levels, surrogate markers are being examined as proxies for cccDNA. Hepatitis B surface antigen (HBsAg) is not a reliable surrogate for cccDNA prevalence or activity, as it is produced from both integrated HBV DNA and cccDNA. Further studies are needed to identify more specific and accurate markers for cccDNA activity and to distinguish between its contributions and those of integrated HBV DNA.

Understanding the differences between cccDNA and chromosomally integrated HBV DNA is crucial for treatment strategies, as their roles in HBV persistence differ significantly. CccDNA serves as the transcriptionally active template for viral replication, sustaining HBV virion production. In contrast, integrated HBV DNA does not contribute to viral replication but can continue to express HBsAg, particularly in HBeAg-negative patients, when cccDNA levels are low or undetectable. This persistent HBsAg production is disease stage-dependent and serves as a long-term antigenic reservoir, even in the absence of active viral replication [8]. In HBeAg-negative patients, integrated HBV DNA becomes a predominant source of HBsAg production, particularly in the later stages of the disease, contributing to ongoing immune evasion [9]. The sustained presence of HBsAg plays a key role in immune suppression by inhibiting toll-like receptor (TLR) activation and impairing both T- and B-cell function, contributing to immune exhaustion and the chronicity of infection [3,10,11]. Despite available therapeutics, maximizing existing public health interventions remains essential in combating HBV. Expanding access to testing, simplifying treatment eligibility criteria, and integrating HBV diagnostics with primary care services have all been identified in the WHO’s 2024 hepatitis B management guidelines. Notably, while NA therapy is effective in reducing HBV-related morbidity and mortality, only 13% of people with CHB are diagnosed, and just 3% receive treatment [3]. These gaps in the cascade of care highlight the urgent need for innovative strategies that not only enhance antiviral efficacy but also improve linkage to care and broaden vaccine coverage. The goal of new therapies for CHB has shifted toward achieving a ***functional cure***, a more attainable objective focused on sustained virological control.

### Defining Functional Cure and Partial Cure

The classic definition of a functional cure was based on observational data from successfully resolved acute HBV infection and from the use of pegylated interferon with and without NA therapy [12,13,14]. This definition included sustained undetectable HBV DNA and HBsAg, along with normal ALT, for at least 24 weeks in the absence of all antiviral therapies (Table 1). This definition has been extensively validated to demonstrate clinical benefits: no development of liver disease and the greatest reduction in risk of HCC relative to untreated chronic HBV infection [15].

The term partial cure has been used to represent persistent but low-level HBV replication reflecting partial immune control (Table 1). In this context, ‘replication’ refers specifically to the conversion of cccDNA-derived pregenomic RNA (pgRNA) into rcDNA, with peripheral HBV DNA serving as a surrogate marker. The definition of a partial cure includes HBV DNA < 2000 IU/mL with normal ALT for at least 6 months in the absence of all antiviral therapies. This definition is based on pegIFN treatment outcomes, where many patients either transitioned naturally from chronic HBV to a partial cure (HBeAg-negative CHB) [16,17] or developed a partial cure (virologic response) after the discontinuation of pegIFN [18,19]. These patients exhibit quiescent liver disease and a reduced risk of HCC (albeit not as low as with a functional cure [20]). However, patients with a partial cure remain at an elevated risk for HBV reactivation [16].

**Table 1:** Definitions of complete (sterilizing), partial, and functional cures.

Type of Cure	HBV DNA Undetectable (U) or <LLOQ		Loss of HBsAg (<0.05 IU/mL)	HBeAg	Normal Levels of ALT	Anti-HBs Seroconversion (Anti-HBs ≥ 10 IU/mL)	Elimination of cccDNA	Year of Definition	Refs.
Complete (Sterilizing)		U	✓	-	✓	+/−	✓	2019	[21]
		U	✓	+/−	✓	+/−	✓	2022	[22]
Functional		U	✓	+/−		+/−		2019	[21]
	<LLOQ		✓	+/−	✓	+/−		2022	[22]
Partial		U	Any	+/−	✓	-		2019	[22]
	<LLOQ		<100 IU/mL					2022	

Positive/negative anti-HB seroconversion indicates that it may or may not occur and is not required for a complete or functional cure. In a partial cure, anti-HB seroconversion is marked as +/−, indicating that it may or may not occur and is not required for a complete or functional cure. In a partial cure, anti-HB seroconversion is always negative.

As clinical data from many approaches (capsid assembly modulators, therapeutic vaccines, TLR agonists, siRNA, and antisense oligonucleotides designed to target HBV mRNA) became available starting in 2016, it became evident that a functional cure was not achieved with these approaches. Given the recognition of the importance of HBsAg loss (and even persistent HBsAg reduction), off therapy [23] modifications to the definitions of a functional cure and a partial cure were proposed (2019 and 2022 endpoint papers). The latest of these proposals includes a relaxed definition of a functional cure, where HBV DNA could be detectable but below the lower limit of quantification (LLOQ) of standard clinical assays. In addition, the latest proposed definition of a partial cure is HBV DNA < LLOQ with HBsAg < 100 IU/mL and normal ALT (Table 1).

The newly proposed definition of a partial cure, which involves HBV DNA suppression below the lower limit of quantification (LLOQ) while HBsAg remains detectable, is not yet validated, and its clinical relevance is uncertain. Although siRNA and antisense-based therapies may help achieve this state, such an outcome is rarely observed, either in the natural progression of chronic HBV infection or after treatment with curative-intent therapies like pegIFN. Additionally, studies on NA withdrawal suggest that maintaining undetectable HBV DNA is only possible when HBsAg loss has also occurred [24]. Even in patients who achieve HBV DNA levels below the limit of detection while on therapy, HBV rebound remains likely if HBsAg is still present after stopping NA treatment [25].

While the protective effect of vaccine-induced anti-HBsAg antibodies (anti-HBs) to prevent the development of chronic HBV infection is beyond doubt, the role of anti-HB seroconversion in establishing a functional cure is not so clear. The production of anti-HBs occurs in all patients with chronic HBV infection but is not detectable as free anti-HBs in most cases are detected with conventional assays [26,27]. However, cases with coexisting free anti-HBs and HBsAg are well described in the literature [28,29], reflecting the genetically diverse nature of chronic HBV infection, where numerous mutations in HBsAg are present which evolved to escape immunological detection [30,31,32]. Thus, the appearance of free anti-HBs during the natural history of chronic HBV infection or during therapy (seroconversion) can either be driven by the efficient elimination of HBsAg or increased immunological function, but the role of anti-HBs is still unclear.

Several studies have suggested a lack of a protective effect of therapeutically induced anti-HBs. The T-cell-adjuvanted HBsAg therapeutic vaccine VBI-2601 (BRI-179) induced anti-HBsAg titers as high as 1000 mIU/mL in monotherapy, with no impact on circulating HBsAg levels, and when combined with pegIFN, VBI-2601 induced anti-HBsAg titers that approached 10,000 mIU/mL, still with no impact on circulating HBsAg [33]. VBI-2601 when added to GalNAc-siRNA (VIR-2218/BRI-835) did not alter HBsAg responses despite the universal appearance of anti-HBs in all patients [34]. During nucleic acid polymer (NAP) therapy, rapid increases in anti-HBs to levels exceeding 200,000 mIU/mL were not accompanied by the formation of HBsAg immunocomplexes during HBsAg clearance, and patients developing anti-HB titers as high as 19,000 mIU/mL experienced the loss of free anti-HBs when HBsAg rebounded during follow-ups [35]. Current clinical data suggest that persistent anti-HBs during follow-ups may be an indicator of the removal of most (if not all) HBsAg-producing capacity in the liver during therapy, which allows free anti-HBs to persist during treatment-free follow-ups.

## 2. Biomarkers and Immune Mechanisms in Achieving a Functional Cure

### 2.1. Virological and Biomarker Predictors of a Functional Cure

Antiviral therapy for HBV impacts a multitude of viral translational products, which can provide markers of treatment responses. Several such markers have been examined as potential indicators of a functional cure [36] (Table 2). However, blood-based markers such as HBsAg and HBV DNA primarily reflect circulating viral components and do not fully capture intrahepatic viral reservoirs, including transcriptionally active cccDNA. Consequently, reliance on peripheral biomarkers alone may underestimate residual intrahepatic viral activity, underscoring the need for additional intrahepatic assessments in therapeutic evaluations.

The accurate assessment of treatment responses and a functional cure requires distinguishing between peripheral and intrahepatic markers, as they represent distinct aspects of HBV biology. Circulating HBV DNA, though originating in hepatocytes, is a peripheral marker that reflects systemic viral replication and the effects of antiviral treatment. Also, intrahepatic markers like cccDNA provide insights into persistent viral reservoirs within hepatocytes. HBV RNA, particularly pgRNA, originates from cccDNA and serves as a surrogate for intrahepatic transcriptional activity [37]. While integrated HBV DNA does not produce pgRNA, it codes for other forms of HBV RNA, leading to potential misinterpretation when using HBV RNA to evaluate treatment efficacy. As a result, peripheral HBV DNA suppression does not necessarily indicate intrahepatic viral clearance, reinforcing the importance of intrahepatic assessments in therapeutic decision-making.

HBeAg-positive and HBeAg-negative stages of chronic HBV infection represent distinct phases of disease progression, each with different viral replication patterns and immunologic characteristics. HBeAg-positive patients typically have higher levels of HBV DNA and active viral replication driven by transcriptionally active cccDNA, whereas HBeAg-negative patients exhibit lower HBV DNA levels due to the emergence of precore and basal core promoter mutations, leading to reduced HBeAg expression and increased immune control over the infection [38]. These differences are clinically significant when evaluating biomarkers, as pgRNA levels decline more rapidly in HBeAg-negative patients undergoing long-term NA therapy than in HBeAg-positive patients. Specifically, a study by Pan et al. demonstrated that HBeAg-negative patients exhibit a steeper reduction in pgRNA-positive rates compared to their HBeAg-positive counterparts, with a higher proportion achieving undetectable levels over time [39]. Given the observed differences in pgRNA kinetics, particularly the more pronounced biphasic decline in HBeAg-negative patients, these findings suggest that the interpretation of ‘cure’ biomarkers such as pgRNA and HBV RNA may require disease-stage-specific thresholds to accurately assess treatment responses.

Hepatitis B core-related antigen (HBcrAg) is a novel biomarker comprising three proteins originating from the precore/core region, including the core antigen (HBcAg), HBeAg, and the pre-core protein [40]. Unlike HBsAg, which can be produced from both cccDNA and integrated HBV DNA, HBcrAg exclusively reflects transcriptional activity from cccDNA, thereby avoiding confounding effects from integrated genomes. However, HBcrAg does not capture transcriptional activity derived from integrated HBV DNA, which can contribute to persistent HBsAg expression despite the suppression of cccDNA activity. Recent evidence further supports the utility of HBcrAg in predicting treatment outcomes.

Huang et al. reported on the results of a cohort of 80 patients who had received NA treatment followed by 96 weeks of PEG-IFNα-based therapy. A sustained response, defined as sustained HBsAg loss ± the generation of anti-HBs 24 weeks after the completion of treatment, was associated with HBcrAg and anti-HBsAg levels. The combination of HBcrAg levels < 4 log_10_ IU/mL and anti-HBs levels > 2 log_10_ IU/L following treatment had a 100% positive predictive value of a sustained response [41].

Carey et al. demonstrated that HBcrAg levels, alongside pre-genomic HBV RNA, can serve as predictive markers for virological relapse following NA withdrawal in HBeAg-negative patients [42]. Specifically, lower HBcrAg levels at treatment cessation were associated with a reduced risk of viral reactivation and ALT flares, underscoring its potential role in guiding treatment discontinuation and assessing the virological control of a therapy. While promising, HBcrAg testing is not yet widely available in routine clinical settings and may be limited by costs [42].

In a study of 222 HBeAg+ patients treated with PEG-IFN ± lamivudine (LAM), those who responded to treatment (HBV DNA < 2000 IU/mL, normal ALT, and HBeAg loss 6 months after treatment) had a decline in both HBsAg and HBcrAg [43]. HBcrAg values < 5.73 log_10_ U/mL at treatment week 24 had a positive predictive value (PPV) for a response of 80%, a sensitivity of 47%, and a specificity of 97%. At week 24 of treatment, HBcrAg values > 8.35 log_10_ U/mL had a negative predictive value (NPV) of 95% for the response. HBcrAg > 8.35 log_10_ U/mL failed to outperform HBsAg values of >20,000 IU/mL as a predictor for nonresponse at week 24 (NPV 100%) [43]. Furthermore, HBcrAg was more frequently detected in patients who failed to maintain HBeAg-negative CHB [5] compared to those who remained in the HBeAg-negative CHB group (29.6% with detectable HBcrAg ≥ 3log_10_ U/mL at baseline versus 6.1% at baseline, respectively) [44].

In another study, 46 patients were treated for 48 weeks with PEG-IFNα. Fifteen patients achieved a virologic response, defined in this study as HBeAg clearance and HBV DNA < 2000 IU/mL with normal ALT 24 weeks after the treatment. Unlike non-responders, patients who responded to the treatment had consistent HBsAg declines. Responders also had greater decreases in HBcrAg than non-responders. HBsAg and HBcrAg levels at week 12 both predicted a response to the treatment. Area under the receiver operating characteristic curve (AUROC) analysis identified a cutoff value of log_10_ 8.0 U/mL for HBcrAg to predict the virologic response at week 12 (a sensitivity of 93.3%, a specificity of 54.8%, a PPV of 50.0%, and an NPV of 94.4%). At 24 weeks, an HBcrAg value of log_10_ 8.0 U/mL had a sensitivity of 100%, a specificity of 35.5%, a PPV of 42.9%, and an NPV of 100% [45].

Factors predicting a sustained response following the removal of NA therapy (defined as HBV DNA < 2000 IU/mL with normal ALT examined at post-treatment weeks 24 and 48) were identified in a cohort of 572 patients who received an average of 295 weeks of therapy. A total of 267 patients achieved a virologic response at the end of the follow-up. Markers predictive of a sustained virologic response included lower HBcrAg (<2 log_10_ U/mL) and HBsAg levels (<50 IU/mL) at the end of treatment [46].

The REP401 study was a phase 2, randomized controlled trial examining NAPs combined with tenofovir disoproxil fumarate (TDF) and PEG IFN-α in HBeAg− chronic HBV patients. Forty participants received 24 weeks of TDF monotherapy, after which they were randomized to receive either 48 weeks of TDF + PEG IFN-α + REP 2139-Mg or REP2165-Mg or 24 weeks of PEG IFN-α + TDF (control therapy) followed by 48 weeks of TDF + PEG IFN-α + REP 2139-Mg or REP2165-Mg. A total of 14 patients achieved a functional cure, 13 attained virologic control, and 1 had experienced viral rebound [47]. The most specific therapy predictor of a functional cure as defined by the REP401 study was an HBsAg decrease to <1 IU/mL with improvements in the predictive value as the HBsAg cutoff was lowered [35]. Other factors predictive of a functional cure included HBsAg monophasic decline [48], HBsAg seroconversion, as well as HBV RNA and HBcrAg both below the lower limit of detection [35].

A separate study analyzed predictors of treatment success in 242 HBeAg-negative patients following 52 weeks of treatment with PEG-IFNα. A response was defined by the loss of HBsAg. The study identified multiple potential markers at baseline, 12 weeks, and 24 weeks. Baseline predictors of HBsAg loss included age ≤ 40 years, ALT ≤ 40 U/L, and HBsAg ≤ 100 IU/mL. Week 12 predictors included ALT ≥ 80 U/L, HBsAg ≤ 50 U/L, and an anti-HBc level  ≤  8.42 signal-to-cutoff ratio (S/CO), and week 24 predictors included ALT ≥  40 U/L, HBsAg level  ≤  0.2 IU/mL, and an anti-HBc level  ≤  8.46 S/CO. These values were then combined to best predict the overall response, with one point being awarded for achieving any cut-off value except HBsAg levels, which was awarded three points. The likelihood of a response could be predicted at any individual time point (baseline, week 12, or week 24). For example, patients with baseline scores of 0–1 had response rates of 13.5%; patients with baseline scores of 2–3 had response rates of 25.9%, and patients with scores of 4–5 had response rates of 63.6%. At 24 weeks of treatment, the same score distribution of 0–1, 2–3, and 4–5 was associated with response rates of 8.6%, 33.3%, and 98.1%, respectively. The scores could also be incorporated into a cumulative total obtained by adding the scores from baseline to week 24. Cumulative scores at week 24 of 0–3 were associated with a response rate of 8.6%, while patients with cumulative scores of 7–10 had an effective response rate of 35.0%, and those with cumulative scores of 11–15 had 96.4% response rates. The authors used baseline and on-treatment scores to generate recommendations on whether to continue or stop the treatment. For example, patients with baseline scores of 0–1, 2–3, and 4–5 had PEG-IFNα treatment recommendations of slight, weak, and strong, respectively, while cumulative scores at week 24 of 0–6, 7–10, and 11–15 carried a recommendation to stop the treatment, a moderate recommendation to continue the treatment, and a strong recommendation to continue the treatment, respectively [49].

Two major studies further highlight the role of PEG-IFN and TDF combination therapy in improving HBsAg loss rates. The first study demonstrated that 9.1% of patients treated with PEG-IFN and TDF for 48 weeks achieved HBsAg loss by week 72, compared to 0% for TDF alone and 2.8% for PEG-IFN alone [50]. This randomized, multinational trial also showed that combination therapy was effective across HBeAg-positive and HBeAg-negative patients, with genotype A having the highest response rates. The second study analyzed a similar cohort and identified a significant predictive marker of the response: an HBsAg decline of >3.5 log10 IU/mL at week 24 [51]. This marker was associated with a positive predictive value of 85% and a negative predictive value of 99% for HBsAg loss, emphasizing the importance of on-treatment monitoring. These findings reinforce the potential of combining PEG-IFN and TDF to achieve a functional cure in CHB and underscore the value of integrating predictive markers into clinical decision-making.

Recent research has examined the potential utility of circulating HBV RNA as a marker for infection, treatment, and prognosis [37]. The combination of serum HBcrAg and HBV RNA levels was identified as a predictor of relapse (HBV DNA > 2000 IU/mL and ALT at least twice the upper limit of normal) after NA therapy, which was stopped after HBeAg seroconversion and HBV DNA < 50 IU/mL for at least 48 weeks. Specifically, no patients who had undetectable HBV RNA and HBcrAg < 4 log_10_ U/mL at the end of the treatment experienced a relapse, while 46.8% of patients with both detectable HBV RNA and HBcrAg over the cutoff value relapsed within 4 years. Medium-risk individuals, defined as those with either a positive HBV RNA and HBcrAg < 4 log_10_ U/mL or undetectable HBV RNA and HBcrAg RNA > 4 log_10_ U/mL, had a 4-year relapse risk of 17.3% [52].

Recent data highlight the limitations of peripheral biomarkers in capturing the full extent of intrahepatic HBV activity. As discussed in a recent paper by Lok et al., while serum biomarkers such as HBsAg and HBV DNA provide useful indicators of treatment responses, they do not fully account for residual viral reservoirs within hepatocytes [53]. This discrepancy is particularly relevant in clinical trials assessing novel antiviral therapies, where liver tissue profiling may offer a more comprehensive evaluation of the virological response. Given these limitations, incorporating intrahepatic assessments alongside serum biomarkers could enhance the predictive accuracy of treatment outcomes [53]. In a study including 62 patients with CHB, a liver biopsy was performed at baseline and at the end of 96 weeks of NA therapy to measure cccDNA. At pre-treatment baseline, there was a better correlation between cccDNA and HBV DNA levels than between cccDNA and HBV RNA levels. HBsAg levels did not correlate with cccDNA levels. While cccDNA was detectable in all patients following 96 weeks of therapy, 37 out of 62 patients were negative for serum HBV RNA and 32 out of 62 patients were negative for serum HBV DNA. An on-treatment decline in cccDNA was most strongly associated with an HBsAg decline but was also associated with declines in HBV DNA and HBV RNA. The HBsAg level was the only marker that correlated with cccDNA following treatment [54].

Other potential markers for cccDNA were identified. In a study of 54 NA-treated patients with liver biopsies at baseline and month 60 of treatment, univariate analysis identified baseline associations between cccDNA and HBV DNA, HBV RNA, HBV DNA plus RNA, HBcrAg, intrahepatic HBV DNA, and the Ishak fibrosis score. Only HBV DNA + HBV RNA remained associated with cccDNA levels in multivariable analysis, which also showed that HBV DNA + HBV RNA was associated with cccDNA declines over the course of the treatment. At month 60 of treatment, only intrahepatic HBV DNA and the Ishak fibrosis score were correlated with cccDNA levels [55].

NAPs combined with TDF and PEG IFN-α were shown to impact HBV RNA levels in HBeAg-negative participants in the REP401 study (detailed above) [47]. Four HBV RNA kinetic patterns were observed. HBV RNA levels either remained at pre-treatment levels, increased followed by the establishment of new elevated HBV RNA levels (only under TDF+ PEG IFN-α therapy), increased transiently, or declined immediately [56]. Failure to reach HBV RNA LLoQ by 16 weeks of NAPs combined with TDF and PEG IFN-α therapy had a negative predictive value of 100% for a functional cure [56].

In a study by Laras et al., both cccDNA and pgRNA were shown to correlate with viral activity, highlighting pgRNA as a marker of HBV replication, particularly as it provides insights into the transcriptional activity of cccDNA. This correlation is significant because cccDNA persists in hepatocytes, serving as a reservoir for the virus even when HBV DNA is suppressed during antiviral therapy [57]. Furthermore, the pgRNA level was found to reflect cccDNA activity even in the setting of long-term NA therapy [58]. Tao et al. recently assessed how pgRNA changed in patients receiving a 96-month course of NA therapy. The median pgRNA for all study participants declined from 6.91 log_10_ IU/mL at baseline to below the lower limit of detection by year 4 of treatment. pgRNA was still detectable in 38/88 patients following 4 years and in 24 patients after 8 years of treatment. Baseline levels of pgRNA ≤ 7.64 log_10_ IU/mL were correlated with HBeAg seroconversion but were not associated with HBsAg seroconversion [59].

In summary, multiple markers and combinations of markers have been evaluated as indicators of a response to therapy for chronic HBV. For example, HBcrAg levels both alone and in combination with other markers were identified as a predictor of a response to treatment. Undetectable HBV RNA and HBcrAg < 4 log_10_ U/mL at end of treatment was associated with a low likelihood of relapse. HBV DNA, HBV RNA, and HBV DNA + RNA were associated with cccDNA levels at baseline, while HBsAg was shown to correlate with cccDNA levels post-treatment. pgRNA is another marker that may correlate with cccDNA levels. More research is needed to evaluate how markers can be combined to predict a functional cure. Table 2 below includes a summary of markers used to evaluate responses to therapy. A further examination of HBV markers can be found elsewhere [36,60].

**Table 2:** Markers assessing responses to therapy.

#	Marker and Time of Measurement	Therapy	Prediction/Importance	Response Rate	Additional Patient Details	Refs.
1	HBcrAg < 4 log10 IU/mL + HBsAb > 2log_10_ IU/mL at end of treatment (EOT)	PEG-IFNα + ETV (48w) followed by PEG-IFNα (48w)	Identifies patients likely to achieve a functional cure + probability of a functional cure at 6 months post-treatment	A total of 21/80 (26.25%) patients were included. All with HBcrAg < 4 log_10_ and HBsAb > 2 log_10_ at end of treatment had a 100% PPV for a sustained response at 6 months.	80 total patients, prior viral suppression with NA	[41]
2	HBcrAg > 8.35 log10 U/mL (Non-responder)/<5.73 U/mL (Responder) (Week 24)	PEG-IFNα ± LAM (52w)	The probability of a sustained response (HBeAg loss + HBV DNA < 2000 IU/mL) at 6 months. HBcrAg > 8.35 U/mL predicts no response, while HBcrAg < 5.73 U/mL indicates a higher response likelihood.	A virologic response in 15/46 patients (32.6%). NPV at week 12/week 24: HBsAg > 20,000 IU/mL (80%/100%), HBcrAg > log_10_ 8.0 U/mL (94.4%/100%).	222 total patients, HBeAg+	[43]
3	HBsAg > 20,000 IU/mL and HBcrAg > log_10_ 8.0 U/mL (Weeks 12 and 24)	PEG-IFNα (48w)	Identifies non-responders to PEG-IFNα at 24 weeks post-treatment	NPV 94.4% (Week 12) and NPV 100% (Week 24).	46 total patients, HBeAg+	[45]
4	HBsAg < 50 IU/mL (EOT)	Long-term NA	Predicts a virologic response post-NA(24w + 48w)	Virologic response: 51% (291/572) at week 24, 41% (206/504) at week 48, and 47.7% (267) at the last follow-up. Re-treatment: Required in 43% (246) of patients after therapy discontinuation. HBsAg < 50 IU/mL at the end of therapy: a total of 64% achieved a virologic response at the follow-up.	572 total patients (24w), 504 (48w), no IFN history, HBeAg (-)	[46]
5	HBcrAg < 2 log_10_ U/mL (EOT)	See Row 4	See Row 4	Virologic response and re-treatment: See Row 4 A total of 65% with HbcrAg < 2 log IU/mL at the end of treatment had a virologic response at the follow-up.	See Row 4	[46]
6	HBsAg < 1 IU/mL (On Tx)	NAP + TDF + PEG-IFNα	Predicts a functional cure (HBsAg loss, normal ALT, and undetectable HBV DNA after 48w)	A total of 14/40 patients achieved a functional cure, while 13/40 patients attained virologic control.	HBeAg− patients, the REP401 study	[35,47,48]
7	Baseline: Age ≤ 40, ALT ≤ 40, and HBsAg ≤ 100 IU/mL | Weeks 12 and 24: ALT, anti-HBc, and HBsAg thresholds	PEG-IFNα (52w)	A scoring system predicting the response likelihood at baseline and weeks 12 and 24	The cumulative score system predicted response rates of 8.6%, 19.1%, 35.0%, and 96.4%.	242 total patients, HBeAg-negative, and anti-HB-negative	[49]
8	HBsAg on NA Therapy	NA (multiple)	Correlation with cccDNA levels	Correlation coefficients: r = 0.66 (HBeAg−) and r = 0.47 (HBeAg ≤ 50 S/CO).	Undetectable serum HBV DNA while on NA therapy, 90 total patients	[61]
9	Undetectable HBV RNA and HBcrAg < 4 log_10_ IU/mL (EOT)	NA discontinuation after seroconversion + HBV DNA < 50 IU/mL for ≥48w	Predicts relapse risk post-NA	A total of 0/14 patients with undetectable HBV RNA and HBcrAg < 4 log_10_ IU/mL relapsed.	127 total patients	[52]
10	Pre-Tx HBV DNA/RNA, and Post-Tx HBsAg	LAM/ADV (96w)	Correlation of HBV DNA/RNA and HBsAg with cccDNA levels	Correlation coefficients: HBV DNA (r = 0.36), HBV RNA (r = 0.25), and HBsAg (r = 0.39).	HBeAg+, ALT ≥ 2× ULN, HBV DNA ≥ 10^5^ copies/mL, and no recent antiviral therapy (≤6 months). A total of 82 total patients at baseline and 62 at 96 weeks.	[49]
11	Baseline and on-treatment HBV DNA + RNA	ETV or ADV	Correlation with cccDNA = identify surrogate markers for cccDNA	Correlation between baseline serum HBV DNA + RNA and cccDNA: β = 0.205 and r = 0.698 Correlation between on-treatment declines in HBV DNA + RNA and cccDNA: β = 0.172 and r = 0.525	HBeAg+, n = 54	[55]

### 2.2. Immune System Dysregulation and Therapeutic Implications in the HBV Functional Cure

A thorough discussion of how HBV impacts the immune system is important for understanding the concept of a functional cure. Indeed, chronic infection is facilitated by ineffective immune responses and functional exhaustion [62]. Unless otherwise specified, the following discussion pertains to CHB infection. A number of immune cells were assessed in patients with CHB, including total T cells; CD4+ T cells; CD8+ T cells; B cells; NK cells; monocytes; regulatory T cells (Tregs); and naïve, central memory (CM), effector memory, and terminally differentiated (EMRA) T cells [63]. In one study, only CD4+ central memory T cells were increased in CHB patients compared to healthy donors, indicating that CHB does not significantly alter the frequency of immune cell response phenotypes regardless of HBsAg levels [63]. A further study found no difference in the frequencies of CD4+/CD8+ T cells with naïve, effector, or memory phenotypes based on HBsAg levels. Moreover, there was no difference in NK cells, B cells, mononuclear phagocytes, mucosal-associated invariant T cells, or Tregs in patients with CHB [64].

New research has provided deeper insights into the intricate interplay between HBV and the immune system. Notably, Bosch et al. demonstrated that HBV infection is accompanied by a marked reprogramming of the hepatic immune microenvironment, influencing both innate and adaptive immune cells to promote viral persistence [65]. Their work specifically highlights how local immune cells in the liver, such as Kupffer cells and liver sinusoidal endothelial cells, can exhibit immunosuppressive phenotypes that restrain antiviral T-cell function and foster T-cell exhaustion. In addition, they observed that HBV-induced signals dampen protective innate responses, thereby contributing to a permissive niche for chronic infection. These findings underscore the critical role of localized immune regulation within the liver, revealing novel pathways through which HBV can evade clearance. Incorporating these insights refines our understanding of immune exhaustion in chronic HBV and suggests that targeting the hepatic microenvironment, alongside systemic approaches, may open up new therapeutic avenues for achieving a functional cure.

Magri et al. further elucidated the role of immune gene expression in HBV persistence [9]. They developed a qPCR method to differentiate between HBV covalently closed circular DNA (cccDNA)-derived and integrant-derived viral transcripts, finding that cccDNA transcription correlates with hepatic inflammatory gene expression rather than integrant-derived transcripts. This study revealed an immune gene signature associated with cccDNA transcriptional activity, reinforcing the concept that inflammation plays a crucial role in regulating viral persistence. Notably, their findings suggest that while integrant-derived viral products, such as HBsAg, contribute to immune exhaustion, hepatic immune activation and inflammation are more directly associated with cccDNA activity. This has important implications for therapeutic strategies aimed at achieving functional cures, as targeting inflammatory pathways and hepatic immune modulation could enhance the effectiveness of HBV-directed therapies.

Recent studies have highlighted the pivotal role of dendritic cell dysfunction in chronic HBV infection, further elucidating the mechanisms of immune evasion. Dendritic cells (DCs) from CHB patients exhibit diminished antigen-presenting capacity and reduced IL-12 production, impairing effective T-cell activation and contributing to inadequate antiviral immune priming. This dysfunction is exacerbated by alterations in monocyte-derived DCs, which favor a tolerogenic environment through increased IL-10 secretion and regulatory T-cell induction [66]. In parallel, intrahepatic immune responses are markedly suppressed in CHB due to the downregulation of interferon-stimulated genes (ISGs) and TLR signaling, including TLR3 and TLR7, leading to impaired IFN-β production. The persistence of high HBsAg levels further dampens TLR-mediated cytokine responses, weakening innate immune activation. These findings underscore the immunosuppressive landscape fostered by HBV, highlighting the need for therapeutic strategies aimed at restoring dendritic cell function, reinvigorating antiviral T-cell responses, and counteracting immune exhaustion to facilitate functional cures [66]. CHB infection causes immune exhaustion that limits the functional ability of the immune system to combat the virus, contributing to viral persistence [62]. Targeting the immune system to augment the immune response provides a potential strategy to achieve a functional cure [67]. Immune exhaustion is largely driven by high levels of subviral particles (SVPs) containing HBsAg, which are produced in excess during CHB and overwhelm the immune system. CD4+ T cells from patients who cleared HBsAg had higher levels of activation markers, including HLA-DR and CD25, than patients who remained HBsAg-positive and healthy controls. HLA-DR was also increased on CD8+ T cells. The exhaustion marker PD-1 was higher on both CD4+ and CD8+ T cells, and CD95 was increased on CD4+ T cells in infected patients compared to healthy controls. Patients who had at least a 30% decrease in HBsAg over 6 months (rapid decrease) had higher levels of HLA-DR and CD-107a on both CD4+ and CD8+ T cells compared to those who did not. Other T-cell markers that were elevated in the rapid decrease group included TIM-3, CD40L, and CTLA-4 on CD4+ T cells and CD69 on CD8+ T cells. HLA-DR, CTLA-4, CD95, CD107a, and TIM-3 expression on CD4+ T cells and HLA-DR and TIM-3 expression on CD8+ T cells predicted HBsAg loss by week 48 of therapy [68]. When comparing patients with high HBsAg levels of >50,000 IU/mL against those with low HBsAg levels of <500 IU/mL, PD-1 expression was higher on CD4+ T cells but not CD8+ T cells. The co-expression of PD-1 and the inhibitory marker 2B4 was also increased in the high-level HBsAg group compared to the low HBsAg group, a difference that was most pronounced in CD4+ EMRA T cells. Notably, the low HBsAg group did not have a statistically significant difference in the expression levels of these inhibitory molecules when compared to healthy controls [63]. This is important because targeting inhibitory receptors such as PD-1:PD-L1 may offer a treatment strategy for chronic HBV. Polyfunctional CD4+ T cells secreting two or more cytokines were more common in the low HBsAg group than the high-level HBsAg group. Core-specific CD8+ T cells secreting both IFNγ and IL12 were more common in the low HBsAg group. There was no difference in IFNγ/TNFα- or IFNγ/IL12-secreting CD8+ T cells. Collectively, these findings suggest that polyfunctional T cells are more common in patients with low HBsAg levels [63]. Intriguingly, HBV-specific IFNγ-secreting CD4+ T cells have been associated with increased viral clearance [69].

Not all studies have observed differences in cytokine production or receptor expression associated with HBsAg levels. However, compelling evidence indicates that HBsAg exerts a suppressive effect on TLR-mediated cytokine responses, particularly at higher concentrations. For instance, Jiang et al. demonstrated that elevated HBsAg levels inhibit TLR-induced production of IL-6 and IFNs, suppressing innate immune responses within the liver [70]. Additionally, TLR3-induced cytokines, such as IFN-α and IFN-β, are markedly suppressed at higher HBsAg levels but show enhanced responsiveness when HBsAg concentrations are reduced, underscoring the graded effects of HBsAg on innate immunity [63]. In a study of 48 persons with CHB, there was no difference in the expression of the immunoregulatory molecules PD-1, CTLA-4, TIM-3, KLRG1, CD160, BTLA, and TIGIT between patients with low HBsAg (<1000 IU/mL) or a high level of HBsAg (>10,000 IU/mL). The lack of observed differences might have resulted from the maintenance of HBsAg levels in both groups above thresholds at which adaptive immune suppression occurs [70]. While the quantitative HBsAg level was correlated with the number of T cells specific for the HBV protein HBs, age was the only significant factor associated with HBs-specific T cells in multivariate linear regression. Indeed, reductions in HBs-specific T cells were noted in patients older than 30 years of age. HBs-specific T cells comprised 30–40% of HBV-specific T cells in patients 3–24 years old but <10% in those older than 35 years. In patients with CHB, T-cell expansion was less robust than those with acute infections, and no IFNγ-secreting HBs-specific T cells were seen in the 11 patients with chronic infection. This contrasted markedly with age-matched, acutely infected patients, in whom HBs-specific T cells comprised approximately 33% of the total T-cell count. The data suggest that the length of exposure to HBsAg and not the quantity of HBsAg results in the deletion of HBs-specific T cells [64].

When assessing T-cell responses, patients who cleared HBsAg exhibited higher frequencies of IFNγ-secreting CD4+ and CD8+ T cells specific for the core antigen than in those who remained HBsAg-positive (1.21% CD4+ and 1.59% CD8+ T cells in patients who cleared HBsAg vs. 0.79% CD4+ and 0.86% CD8+ in HBsAg-positive patients). Furthermore, the degree of HBsAg reduction was positively correlated with HLA-DR, CD95, CD40L, CTLA-4, TIM-3, and CD107a on CD4+ T cells and HLA-DR, CD69, and CD107a on CD8+ T cells. The CD8+ T-cell expression of HLA-DR and CD95 was associated with the magnitude of the CD8+ T-cell response to HBcAg, and PD-1 expression on CD4+ T cells and granzyme B expression, a marker of T-cell effector function, on CD8+ T cells was correlated with the strength of the response to HBsAg [68].

PD-1 was shown to be upregulated in CHB [71], and PD-1:PD-L1 axis manipulation has gained attention as a potential treatment method with the goal of augmenting the immune response to HBV [72]. The ex vivo response to the PD-L1 inhibitor MEDI2790 was assessed. Responders, marked by a minimum increase of 10 spot-forming units (SFUs) in ex vivo ELISpot assays, had a higher frequency of TIM-3 + PD-1 + PD-L1+ T cells without LAG3 expression. Nonresponders had a higher proportion of functionally exhausted T cells with at least three inhibitory markers (LAG3 +, TIM3 +, and PD-1+). The authors concluded that LAG3 expression impairs responses to the PD-L1 blockade. Importantly, the PD-L1 blockade improved the HBV-specific T-cell response; the PD-L1 blockade increased cytotoxicity and IFNγ production in HBV-specific T cells. IL-10 production also increased [73]. The PD-L1 blockade may be enhanced by the addition of IL-15. Specifically, the combination of the two can ameliorate CD8+ T-cell responses targeting core peptides in patients with T-cell exhaustion. (SAT-374-YI IL-15 plus anti-PDL-1 restores CD8+ T-cell responses against the core antigen but not against polymerase in CHB with extreme exhaustion-associated factors [74].)

Given that some studies have demonstrated PD-1 upregulation in chronic HBV, the efficacy of PD-1 inhibition in contributing to a functional cure via the restoration of normal immune function has been assessed [75]. Patients were treated with the PD-1 inhibitor nivolumab at either 0.1 mg/kg (n = 2) or 0.3 mg/kg (n = 22). Ten patients in the 0.3 mg/kg group also received the therapeutic HBV vaccine GS-4774. While the 0.1 mg/kg group did not have any significant difference in HBsAg levels at 12 weeks, 20/22 patients treated with 0.3 mg/kg of nivolumab ± GS-4774 had lower HBsAg levels (9/10 patients who received GS-4774 and 11/12 who did not receive GS-4774). The two 0.3 mg/kg nivolumab groups combined showed an average HBsAg decrease of 0.48 log_10_ IU/mL by week 24. The one patient who achieved HBsAg loss demonstrated the strongest T-cell response, as assessed by IFNγ and TNFα FluoroSpot. When analyzing all 24 patients, there was a decrease in activated, CD39-expressing Tregs by week 24. There were fewer naïve CD4+ T cells and greater numbers of CD4+ CCR7-CD45RA- effector memory cells. Furthermore, there were fewer naïve CD8+ T cells and more CCR7-CD45RA- effector memory, CCR7-CD45RA+ effector memory RA, CD56+, and CD57+ CD8+ T cells at week 24 [75].

In a separate study, the immunologic profile of 80 patients who received 96 weeks of PEG-IFNα therapy was assessed [41]. While HBV-specific CD8+ T cells, total B cells, memory B cells, and CD86/CD95 B-cell expression were stable in patients who lost HBsAg and generated anti-HBs, patients who did not respond had decreased proportions of CD8+ T cells specific for the core and envelope as well as decreased CD86 and CD95 expression on B cells. Patients with HBcrAg < 4 log_10_ U/mL or anti-HBs > 2 log_10_ IU/L following treatment (values predictive of a durable response to treatment) had stable to increased levels of HBV-specific CD8+ T cells. Patients achieving HBcrAg < 4 log_10_ U/mL had increased follicular helper T (Tfh) cells. B-cell proportions and CD86 (a B-cell costimulatory molecule) expression [76] were stable in patients in the previously mentioned two subgroups (HBcrAg < 4 log_10_ U/mL or anti-HBs > 2 log_10_ IU/L following treatment). B-cell proportions were decreased in those with an anti-HB level of ≤ 2 log_10_ IU/L following treatment. These findings indicate the maintenance of both humoral and cellular immunity in patients achieving the cutoff values for HBcrAg and anti-HBs [41]. This study elucidates changes in the T-cell compartment that are associated with a response to treatment, including alterations in Tfh cells. A more complete understanding of these changes might facilitate the development of treatments that could contribute to a functional cure [41].

The T-cell response was characterized following treatment cessation in 27 patients with HBeAg-negative HBV with at least 3 years of virologic control on NA. Patients with a durable response had HBV-specific CD8+ T-cell responses against multiple HBV proteins prior to completing the NA treatment. A T-cell response, which was assessed by in vitro stimulation with overlapping peptides including the core, polymerase, and envelope proteins, was defined as a response of at least 0.1% following the subtraction of the unstimulated control. Patients who remained off treatment were more likely to have CD4+ and CD8+ T cells responding to all HBV antigens. In fact, IFNγ- and TNFα-producing cells responding to any peptide pool were not seen in patients who relapsed [77]. T-cell degranulation following a challenge with core protein overlapping peptides was more frequent in patients with a durable response, but similar results were not observed following challenges with overlapping peptides from envelope and polymerase proteins [77]. The group that remained off treatment also had a higher proportion of polyfunctional CD8+ T cells co-producing IFNγ and TNFα. Notably, baseline virologic serum and intrahepatic markers, including HBcrAg, intrahepatic HBV DNA, intrahepatic HBV RNA, cccDNA, and the intrahepatic HBV RNA/cccDNA ratio, did not correlate with CD4+ or CD8+ T-cell response strength [77]. A similar analysis was performed in 15 HBeAg-negative patients who had received NA therapy for at least 3 years. Patients with chronic HBV had alterations in their T-cell compartment compared to healthy individuals. For example, HBV patients had altered expression levels of several markers such as PD-1 and CCR7 on T cells. They also had more differentiated T cells as well as an increased frequency of central and effector memory T cells. NA discontinuation had a very small impact on T-cell phenotypes. Following treatment, there were more core-specific IFNγ+ and MIP-1β+ CD4+ T-cell responses, and the frequency of IFNγ-secreting CD8+ T cells was increased. Multifunctional T-cell responses to core antigens were also increased. Similar results were not seen with envelope or polymerase antigens. Notably, this augmented response to core peptides was not consistent in all patients. Clinical data and HBsAg loss did not necessarily correlate with the strength of the T-cell response [78].

IFNα therapy does have an impact on the T-cell phenotype, as seen with NA discontinuation [79]. Patients treated with IFNα had higher expression levels of HLA-DR and CD25 on CD4+ T cells and higher levels of HLA-DR on CD8+ T cells than those who received NA or no treatment. Compared to patients treated with NA, the IFNα group had higher CD4+ T-cell expression of CTLA-4 and CD8+ T-cell expression of CD69. Both treatment groups had higher PD-1 expression, although there was no difference in the HBcAg-specific response frequency [68]. Collectively, these studies indicate how the treatment influences the T-cell compartment. A better understanding of these changes could facilitate the development of novel avenues to predict treatment success.

A thorough understanding of B-cell changes mediated by HBV is important to identify potential treatments that may contribute to a functional cure. Indeed, therapies targeting the B-cell compartment are already being examined [80]. In 27 patients with CHB, HBcAg-specific B cells were identified in 26 patients, while HBsAg-specific B cells were much less common [81]. Similar findings have been documented in other studies, where HBsAg-specific B cells were 17.8-fold less frequent than HBcAg-specific memory B cells. This is consistent with the idea that the humoral response against HBV is targeted towards HBcAg rather than HBsAg [82]. However, B cells exhibit functional defects in persons with CHB, including impaired antibody production and differentiation into plasma cells, as well as increased expression of inhibitory receptors such as PD-1 on atypical memory B cells (atMBCs). An accumulation of atMBCs in patients with CHB dampens antiviral responses and contributes to viral persistence [83]. HBcAg-specific B cells were much less common in patients who had cleared an HBV infection. While HBcAg-specific B cells could mature into antibody-secreting B cells in the presence of IL-2, IL-12, and fibroblasts expressing the CD40 ligand, this did not occur in HBsAg-specific B cells, even following the administration of the innate immune stimulus CpG [81]. Chronic HBV resulted in an increase in total atMBCs, a change particularly noted in HBsAg-specific B cells. HBcAg-specific B cells did not have a significantly elevated frequency of atMBCs. In addition, HBcAg-specific B cells tended to be IgG+ with higher levels of the activation marker CD95 and lower levels of the inhibitory marker IL10-Rα. HBsAg-specific B cells tend to be IgM+. Notably, both populations had differential gene expression when compared to the global B-cell population, including the upregulation of genes involved in dendritic cell recruitment and innate immune activation [81]. This is similar to findings demonstrating increased levels of inhibitory markers on HBs+ memory B cells (SAT-399 immune profiling of HBsAg-specific B cells in CHB patients with HBsAg loss) [84]. HBsAg levels had a slight impact on B-cell receptor expression. Markers for immune B-cell exhaustion including FcRL4, FcRL5, PD-1, and PD-L1 were assessed in the setting of high HBsAg levels (>50,000 IU/mL) and low HBsAg levels (<500 IU/mL). FcRL5 was the only receptor that was differentially expressed; this receptor was more common in the high HBsAg group [63]. Other studies have demonstrated significant effects on B-cell function in the setting of chronic HBV infection. Indeed, B cells from chronic HBV patients demonstrated 939 genes that were upregulated, including increases in inhibitory markers such as Fc family receptors, and 968 genes that were downregulated, including genes involved in antigen presentation and B-cell receptor signaling, compared to the control [85]. Atypical B cells, which are thought to be exhausted/anergic cells whose phenotype is generated during chronic antigen stimulation, often express inhibitory Fc family receptors [86,87]. Consistent with this, atypical B cells were more common in chronic HBV. These cells demonstrated a dysfunctional HBs-specific response. Moreover, CXCR5^hi^CD4Tfh subsets were seen at higher frequencies in HBV patients compared to healthy controls. These cells expressed CD40L, raising the possibility that antigen presentation coupled with Tfh-mediated CD40L resulted in the generation of atypical memory and naïve B cells. Furthermore, incubating PBMCs from healthy individuals with HBc stimulated FcRL4 and FcRL5 expression in a dose-dependent fashion. PD-1 was also upregulated by HBV antigen-mediated signaling and CD40L. FcRL5 was upregulated on PD-1+ cells. Mechanistically, it was shown that HBc stimulated B-cell proliferation, particularly in FcRL5+ cells [85]. Collectively, these studies on B cells show how chronic HBV alters the B-cell compartment, specifically by inducing the expression of markers associated with B-cell exhaustion, changing gene expression, and altering phenotypes. These changes pose potential therapeutic targets.

In summary, HBV has a multitude of effects on the B- and T-cell compartments, including but not limited to changes to cell marker expression, increased cell exhaustion, and impacts on gene expression. Additionally, certain immune profiles are associated with an increased likelihood of HBsAg loss. Examples include increased expression of activating markers such as HLA-DR, CTLA-4, and CD25 on T cells and higher frequencies of IFNγ-secreting CD4+ and CD8+ T cells specific for the core antigen. By contrast, T-cell exhaustion is associated with progressive infection, and other changes such as fewer CD8+ T cells targeting the HBV core and envelope proteins and changes in the B-cell compartment such as decreased CD86 and CD95 expression were associated with an inability to clear HBsAg. While much focus has been placed on the adaptive immune response, the innate immune system also plays a critical role in HBV immune control. NK cells and pattern recognition receptor (PRR) pathways contribute to early viral recognition, immune priming, and the modulation of antiviral responses. Table 3 summarizes some of the mechanisms through which HBV impacts the immune system.

**Table 3:** Mechanisms of HBV’s impact on the immune system.

Item #	Viral Component/Treatment Impacting the Immune System	Effect of Viral Component/Treatment on Immune System	Citation
1	Effect of chronic HBV (persistence of HBsAg for at least 6 months) on T cells	- Alterations in T-cell markers, more differentiated T cells, and more central and effector T-cell in chronic HBV patients on long-term NA therapy compared to healthy individuals. - Increased CD4+ central memory T cells. - A minimum change in the T-cell phenotype based on HBsAg levels.	[64,78]
2	HBsAg level > 50,000 vs. < 500 IU/mL	Expression of FcRL5 on T and B cells with HBsAg > 50,000 IU/mL.	[63]
3	HBsAg level > 50,000 vs. < 500 IU/mL	Higher polyfunctional T-cell frequency with HBsAg < 500 IU/mL.	[63]
4	At least a 30% decrease in HBsAg over 6 months of IFN therapy (rapid decrease) vs. those who did not have a rapid reduction in HBsAg	Alterations in multiple T-cell markers, including higher HLA-DR and CD-107a on CD4+ and CD8+ T cells; TIM-3, CD40L, and CTLA-4 on CD4+ T cells; and CD69 on CD8+ T cells.	[68]
5	HBsAg clearance	Increased T-cell expression of activation markers	[68]
6	HBsAg clearance	Higher frequencies of IFNγ-secreting CD4+ and CD8+ T cells specific for the core.	[68]
7	HBcrAg < 4 log_10_ U/mL and HBsAb > 2 log_10_ IU/L following treatment, which is associated with a sustained response as defined by HBsAg loss +/− HBsAb at 24 weeks post-treatment	Sustained humoral immunity and stable/increased levels of HBV-specific CD8+ T cells.	[41]
8	HBcrAg < 4 log_10_ U/mL post-treatment	Increased Tfh cells.	[41]
	Durable response to NA	Patients with a durable response to NA were more likely to have HBV-specific CD8+ T-cell responses against multiple HBV proteins prior to completing NA treatment.	[77]
	NA discontinuation	- IFNγ- and TNFα-producing cells responding to any peptide pool were not seen in patients who relapsed after attaining 3 years of - virologic control on NA therapy. - NA discontinuation had only a small impact on the T-cell phenotype. - HBsAg loss and the strength of the T-cell response did not correlate.	[77,78]
	Effect of chronic HBV (persistence of HBsAg for at least 6 months) on B cells	- HBsAg-specific B cells do not mature to antibody-secreting cells following co-culture with IL-2, IL-21, and fibroblasts expressing CD40L. HBc-specific B cells maintained the ability to mature and produce anti-HBc. - Increased AtMBC, particularly HBsAg-specific B cells. - Alterations in B-cell gene expression, including dendritic cell recruitment and innate immune activation, as well as increased expression of inhibitory markers on HBs+ memory B cells. - Differential B-cell gene expression, including Fc family receptor upregulation and the downregulation of antigen presentation genes, as well as increased atypical B-cell frequency.	[81,85]
9	Effects of HBsAg on innate immunity and B-cell function	- Suppresses TLR-mediated cytokine responses (e.g., IL-6, IFN-α, and IFN-β) at higher concentrations, reducing innate immune activation. - Increases the expression of inhibitory receptors (e.g., PD-1, FcRL4, and FcRL5) on B cells, particularly at high HBsAg levels. - Alters B-cell gene expression, thereby upregulating markers of exhaustion and downregulating antigen presentation pathways. - Promotes atypical memory B-cell accumulation, impairing antibody production and plasma cell differentiation.	[70] [63,81] [85] [83,85]

## 3. Achieving a Functional Cure with Antiviral Therapy

Spontaneous HBsAg loss occurs in approximately 1% of CHB patients annually. Predictive factors include HBeAg−, older age, and undetectable HBV DNA. However, most patients cannot rely on spontaneous clearance to achieve a functional cure. Consequently, the focus has shifted towards leveraging antiviral therapies to enhance the rate of HBsAg loss and sustained virological responses to reach a functional cure. Although reductions in peripheral HBV DNA are commonly used as markers of viral suppression, they do not fully reflect intrahepatic viral activity. Persistent low-level replication, even in individuals on long-term therapy, contributes to ongoing liver disease progression and the continued risk of HCC, reinforcing the need for more effective antiviral strategies.

Given these limitations, new direct-acting antivirals (DAAs) with enhanced potency against HBV replication remain a critical goal. Block et al. highlighted that suppressing intrahepatic viral reservoirs, particularly cccDNA and integrated HBV DNA, is essential to mitigate ongoing hepatocyte turnover and oncogenesis. Current treatment guidelines continue to stratify patients based on HBeAg status, as HBeAg-positive individuals, particularly those in the immune-tolerant phase, have lower rates of viral suppression with existing NAs. The continued use of HBeAg status in treatment decision-making underscores its clinical relevance in predicting treatment responses and long-term prognoses [88].

This section explores emerging strategies aimed at enhancing HBsAg loss, particularly those that go beyond peripheral viral suppression to address intrahepatic viral reservoirs. By integrating novel therapeutic approaches with a better understanding of virological markers, we aim to refine the path toward achieving a functional cure for CHB.

### 3.1. PEG-IFNα Therapy

The S-Collate study examined the efficacy of PEG-IFNα-2a in 844 HBeAg-positive patients and 872 HBeAg-negative patients, of whom 540 and 614 completed the follow-up. A total of 16 HBeAg-positive and 41 HBeAg-negative patients experienced HBsAg loss at the 3 year follow-up mark (5% and 10%, respectively, in patients for whom data were available at the 3 year mark). In HBeAg-positive patients, HBsAg clearance was most likely in patients with week 12 HBsAg levels of <1500 IU/mL, while HBsAg clearance was associated with a ≥10% decrease in HBsAg levels at week 12 in HBeAg-negative patients [89].

In another clinical trial involving treatment-naive HBeAg-positive patients receiving peg-IFNα monotherapy for 48 weeks, the HBsAg loss rate was ~3% (8/271). At the end of a 24-week treatment-free follow-up, HBV DNA was <100,000 copies/mL in 86 of these patients [90]. In HBeAg-negative patients, a study demonstrated that 11 out of 43 patients achieved HBsAg loss 48 weeks after completing IFN therapy [91]. Patients with genotypes A and B had higher on-treatment declines in HBsAg than patients with genotypes C and D [92,93]. A total of 4/16 patients with HBeAg-negative disease cleared HBsAg by 48 weeks post-treatment; however, these patients had significantly lower baseline HBsAg and HBV DNA levels. This study reported that patients with HBsAg < 120 IU/mL and HBV DNA < 5 log_10_ copies/mL may clear HBsAg with PEG-IFNα therapy [94]. In 44 patients receiving PEG-IFNα after failing NA therapy, 7 patients were HBsAg-negative at the last follow-up [95].

### 3.2. Combination and Switching Between PEG-IFNα and NA

In a study of 197 HBeAg+ patients with at least 2 years of NA treatment, 81 patients received PEG-IFNα in addition to ongoing NA. The control group continued NA monotherapy. A total of 50 individuals from each group were included in match-pair analysis. Only 2/100 patients, both in the PEG-IFNα group, cleared HBsAg by week 48. Values for both HBeAg and HBsAg decreased more in the combined therapy group than the NA monotherapy group. In a multivariate analysis of the entire dual therapy study population (81 total patients), lower baseline values for HBsAg were associated with HBsAg clearance. In the entire dual therapy population of 81 patients, HBsAg loss was more frequent in patients with a baseline HBsAg of <1000 IU/mL (7/22 v. 2/59) than those who had a decrease of ≥0.5 log_10_ IU/mL by week 12 (7/19 v. 2/62). A baseline HBsAg of <1000 IU/mL in combination with a decrease in HBsAg of ≥0.5-log_10_ IU/mL by week 12 yielded the best prediction of HBsAg loss [96].

PEG-IFNα was studied in combination with NAs. Patients who had undetectable HBV DNA and were HBeAg− for at least one year on NA therapy and who had no changes to their NA regimen for at least three months were randomized to NA + PEG-IFNα for 48 weeks (n = 90) or continued NA monotherapy (n = 93). HBsAg loss was reported in 7.87% (7/90) patients in the combination therapy group compared to 3.2% (3/93) in the NA monotherapy group (difference 4.6%, CI −2.6 to 12.5, and *p* = 0.15). PEG-IFNα and NA combination therapy did not significantly augment HBsAg clearance over NA monotherapy in this patient population, and combination therapy was associated with more side effects [97].

Switching from NA to IFN therapy once HBV DNA is suppressed was evaluated as a strategy to attain HBsAg clearance [98]. In one study, 88 patients with baseline HBeAg < 100 S/CO who were treated with NA for at least 48 weeks were randomized to either continue with NA therapy (n = 44, 27 HBeAg+) or switch to IFN (n = 44, 29 HBeAg+). There were greater decreases in HBsAg levels in the group that transitioned from NA therapy to IFN therapy (HBsAg levels after 48 weeks of treatment: 3.1340 log_10_ IU/mL vs. 3.6950 log_10_ IU/mL). Seven (5 HBeAg+ and 2 HBeAg−) of 44 patients who received IFN had HBsAg loss, and one had seroconversion, while no patients who remained on NA had HBsAg loss. One patient in the IFN group experienced an HBsAg recurrence during week 24 of the follow-up [99]. Similar results were found in a separate clinical trial that examined the switch from NA to PEG-IFNα, where 8.5% of 94 HBeAg+ patients (HBeAg < 100 PEIU/mL) who received PEG-IFNα had HBsAg loss compared to 0% of 98 patients who remained on NA. Similar to a previous study [99], an early HBsAg decline predicted future responses at the end of treatment [98]. It is important to note that the studies cited in this section did not assess the superiority of NA followed by IFN compared to initial IFN therapy, so it is not clear whether sequential therapy offers an advantage over IFN alone in achieving HBsAg loss.

### 3.3. Long-Term NA Therapy

Third-generation NAs, such as ETV, TDF, and tenofovir alafenamide (TAF), have shown varying efficacy in achieving HBsAg loss. Among HBeAg-positive patients, ETV treatment achieved a 2% HBsAg loss rate at one year, increasing to 3% after five years. TDF treatment was associated with a 3% rate of HBsAg loss at one year and 5% at ten years. TAF treatment had a 1% HBsAg loss rate at one year, increasing to 4% after three years. In contrast, in HBeAg-negative patients, ETV showed no HBsAg loss at one year but increased to 3% after ten years. TDF demonstrated a 0% HBsAg loss rate at one year, rising to 3% at ten years. TAF also showed no HBsAg loss at one year and 3% after three years [100,101]. These findings suggest that while HBsAg loss can occur with prolonged NA therapy, the rates remain low, particularly in HBeAg-negative patients.

In a study of 5409 patients who were initially treated with lamivudine (LAM) or entecavir (ETV) for a median treatment duration of 4.3 years (an IQR of 2.1–6.8 years) and followed for a median of 6 years, 110 patients achieved HBsAg loss, corresponding to a 0.33% annual loss rate. Among the 110 patients who achieved HBsAg seroclearance, 55 (50%) were HBeAg-positive at baseline, compared to 3546 (66.9%) of the 5299 patients who did not clear HBsAg (*p* = 0.01). This indicates that HBsAg seroclearance was significantly more likely in HBeAg-negative patients. A baseline ALT level of >5 times the upper limit of normal was associated with HBsAg loss. Conversely, HBeAg positivity, high HBV DNA levels, and cirrhosis were inversely associated with HBsAg loss. Additionally, 18 patients experienced HBsAg and HBV DNA relapse, which was usually transient and did not require re-treatment. The cumulative rates of HBsAb seroconversion at 1, 2, 3, and 4 years after HBsAg loss were 34.3%, 40.3%, 54.0%, and 67.4%, respectively [102]. In an even larger study including 20,263 CHB patients who received ETV and/or TDF for at minimum 6 months between 2005 and 2016, the HBsAg loss rate was 2.1% after a median follow-up of 4.8 years (IQR: 2.8–7 years) [103] In clinical practice, NAs are often favored over PEG-IFNα because they are administered orally, have limited side effects, and are highly effective in inhibiting viral replication [104].

### 3.4. NA Discontinuation Therapy Approach

Some studies have observed that stopping NA therapy can be associated with HBsAg loss [105]. Modest HBsAg loss rates following NA discontinuation have been reported in HBeAg-positive patients, with median rates ranging from 2% at 24 weeks to 11% at 192 weeks, according to a systematic review of 25 studies. However, other studies observed no HBsAg loss following NA cessation [106]. A notable exception is one study that reported an 8-year cumulative HBsAg loss rate of 19.6% in HBeAg-positive patients who discontinued nucleoside analog therapy (either lamivudine or entecavir). However, the study did not find a significant difference in HBsAg loss rates between patients treated with lamivudine versus entecavir [107]. These findings suggest that although some HBeAg-positive patients may achieve HBsAg loss after discontinuing NA therapy, the rates are generally low and vary across different studies [108].

In contrast, HBeAg-negative patients showed higher rates of HBsAg loss following NA discontinuation. Hadziyannis et al. reported an impressive 39.4% HBsAg loss rate in patients treated with adefovir (ADV), while Berg et al. found a 19% HBsAg loss rate in patients after the discontinuation of TDF [105,109]. Other studies, such as those by Papatheodoridis et al. and Chen et al., also reported notable HBsAg loss rates of 25% and 33.1%, respectively [107,110]. These higher rates in HBeAg-negative patients may be attributed to factors such as longer treatment durations, patient adherence, and lower baseline HBsAg levels, which have been identified as predictors of successful HBsAg loss [108].

In a randomized control trial (RCT) of non-cirrhotic HBeAg-negative patients (n = 42), participants who had been receiving TDF for at least four years with suppressed HBV DNA for a minimum of 3.5 years were assigned to either discontinue (Arm A, n = 21) or continue (Arm B, n = 21) TDF monotherapy. Follow-ups were conducted through week 144 post-treatment to explore the potential for a sustained virological response after stopping long-term TDF therapy under strict surveillance [109]. Of the 21 patients who discontinued TDF (Arm A), 13 (62%) remained off therapy for the entire 144-week follow-up period. Notably, within this subgroup, 43% (9 out of 21 in arm A) achieved either HBsAg loss or an HBV DNA level below 2000 IU/mL. The findings suggest a potential for sustained virological responses following the discontinuation of long-term NA therapy [109]. This finding was confirmed by a larger study that included 1075 HBeAg-negative patients treated with TDF for a median of 156 weeks (61–430 weeks). During treatment, only five patients (0.15% annual incidence) had HBsAg loss. After stopping NA therapy, 691 patients (median age 52.3 and 86% male, with 44.6% having cirrhosis) were prospectively followed up. Over a median off-therapy time of 155 weeks, 42 patients (6-year cumulative incidence of 13% and annual incidence of 1.78%) achieved HBsAg loss. Factors associated with HBsAg loss after the discontinuation of NA included shorter times to undetectable HBV DNA (<12 weeks), greater HBsAg reduction during therapy (>1 log_10_), lower end-of-treatment HBsAg levels (<100 IU/mL), sustained responses, and patients with clinical relapse not receiving re-treatment. Patients with clinical relapse who remained off treatment had a 7.34 times higher incidence of HBsAg clearance than those who resumed treatment. The study highlights a much higher incidence of HBsAg loss after stopping NA therapy compared to during treatment, emphasizing the potential impact of transient untreated clinical relapse on immune control [111].

A meta-analysis of 17 studies was performed to identify predictors of relapse after the withdrawal of NA therapy in patients who were HBeAg-negative. The predictors of virological relapse were age (HR = 1.022 per year), baseline HBsAg level (HR = 1.509 per log_10_ IU/L), end-of-treatment (EOT) HBsAg level (HR = 1.896 per log_10_ IU/L), an EOT HBsAg level of ≥1000 IU/mL (HR = 1.749), and HBsAg decline from baseline to EOT (HR = 0.748 per log_10_ IU/L). Regarding clinical relapse, baseline HBsAg levels (HR = 1.312 per log_10_ IU/L), EOT HBsAg levels (HR = 1.458 per log_10_ IU/L), an EOT HBsAg level of ≥100 IU/mL (HR = 3.199) or ≥1000 IU/mL (HR = 1.810), and the duration of consolidation therapy (HR = 0.991 per month) emerge as predictive factors. The findings suggest that older age, a shorter duration of consolidation therapy, and higher HBsAg levels at baseline and EOT predict relapse after the discontinuation of NA in HBeAg-negative patients [112]. Additionally, another study demonstrated that the cumulative probability of HBsAg loss was 3.2% at 12 months and 13.0% at 48 months, with higher HBsAg loss in Caucasians compared to Asians. Specifically, Whites with HBsAg levels of <1000 IU/mL at the end of therapy had a significantly higher predicted probability of HBsAg loss compared to Asians, who showed a higher probability when their HBsAg levels were <100 IU/mL. This study underscores the importance of considering patient ethnicity and HBsAg levels at the end of therapy when predicting HBsAg loss and making clinical decisions regarding NA withdrawal [113].

### 3.5. Drugs in Development

#### 3.5.1. HBsAg Reduction

##### Bepirovirsen

A dose-finding study of bepirovirsen, a dual function unconjugated oligonucleotide drug with both immunostimulatory and antisense properties, randomly assigned 457 participants to four groups in a 3:3:3:1 ratio: group 1 received weekly subcutaneous injections of bepirovirsen at a dose of 300 mg for 24 weeks; group 2 received the same dose for 12 weeks followed by 150 mg for another 12 weeks; group 3 received 300 mg for 12 weeks followed by a placebo for 12 weeks, and group 4 received a placebo for 12 weeks followed by bepirovirsen at a dose of 300 mg for 12 weeks. Loading doses of bepirovirsen were administered in groups 1, 2, and 3. A composite primary outcome was defined as maintaining an HBsAg level below the limit of detection and an HBV DNA level below the limit of quantification for 24 weeks after the end of treatment, without initiating new antiviral medication. Bepirovirsen dosed at 300 mg per week for 24 weeks resulted in HBsAg loss in 28% of participants at EOT; however, this declined to 9–10% at 24 weeks post-treatment, indicating a relatively rapid rate of rebound. The follow-up period of 24 weeks is relatively short and limits conclusions regarding the long-term durability of the response. Responses were primarily observed in participants with low baseline HBsAg levels. Bepirovirsen’s off-target immunostimulatory effects seem to play an important role in the observed declines in HBsAg levels during therapy, particularly in patients with low baseline HBsAg levels [114].

##### SiRNA-JNJ-3989

JNJ-3989 consists of two GalNAc-conjugated short-interfering RNA (siRNA) triggers designed to target HBV RNA produced from both cccDNA and HBV DNA integrated into the host genome to reduce HBsAg levels. This compound was evaluated in combination with NAs, with or without the capsid assembly modulator (CAM) JNJ-6379, in a recent phase 2a study [115]. The study included 84 patients, both treatment-naïve and NA-suppressed, who received JNJ-3989 in doses ranging from 100 to 400 mg every 4 weeks, along with daily NA treatment. HBsAg decreased by >1 log_10_ IU/mL from the initial baseline in 39 out of 40 patients, accounting for 97.5% of the participants in this dose range. Moreover, all 12 patients receiving the triple combination of JNJ-3989, NA, and JNJ-6379 also had a >1 log_10_ IU/mL reduction in HBsAg, including HBeAg-positive and HBeAg-negative patients. Notably, HBsAg reductions of >1 log_10_ IU/mL persisted in 38% of patients 336 days after the final JNJ-3989 dose. However, most patients exhibited a rebound toward baseline levels during the follow-up, consistent with the pharmacodynamics of GalNAc-siRNA therapies [115]. A phase 2b study further explored the efficacy and safety of JNJ-3989 and JNJ-6379 in non-cirrhotic (F0-F2), HBeAg-negative patients on NA treatment. One hundred and thirty patients were randomized to receive JNJ-3989 and JNJ-6379 with NA (active arm) or a placebo with NA (control arm) for 48 weeks. The primary endpoint was HBsAg loss 24 weeks after completing therapy, without restarting NA treatment [116]. No patients achieved the primary endpoint of HBsAg loss at follow-up week 24 in either treatment arm. However, the active arm displayed a notable reduction in HBsAg levels during treatment, with 71.1% of patients achieving HBsAg levels below 100 IU/mL at week 48. By follow-up week 48, 46.9% of patients in the active arm maintained HBsAg levels < 100 IU/mL, compared to 15.0% in the control arm. However, mean HBsAg levels steadily increased during the follow-up in most patients, indicating that these reductions reflected transient suppression rather than durable off-treatment control [116]. Updated results demonstrated that 81.5% of patients in the active arm had reductions in HBsAg from baseline (>1 log_10_ IU/mL) to follow-up week 48, compared to 12.5% in the control arm. The mean HBsAg decline in the active arm (−1.89 log_10_ IU/mL) was significantly greater than in the control arm (−0.06 log_10_ IU/mL). Additionally, 9.1% of patients in the active arm and 26.8% in the control arm restarted NA during the follow-up due to HBV DNA relapse and ALT increases [117]. It should be noted that one of the patients who was previously maintained on NA therapy with undetectable HBV DNA levels experienced a flare that progressed to subacute liver failure, which necessitated an emergency liver transplant. This incident underscores the potential risks associated with discontinuing NA treatment and highlights the need for careful monitoring in clinical trials exploring new HBV therapies [118].

Preliminary data for another GalNAc-siRNA agent, VIR-2218, in combination with PEG-IFNα, were reported in an abstract form. The study demonstrated that 30% of participants achieved HBsAg loss at the end of treatment, and 16% maintained sustained HBsAg loss 24 weeks post-treatment [119]. However, the study had notable limitations, including the absence of a PEG-IFNα monotherapy control group, complicating the interpretation of these findings, as PEG-IFNα alone is known to achieve a functional cure in approximately 30% of patients [120,121,122,123]. Persistent HBsAg loss with the combination was only observed in participants with very low HBsAg levels, emphasizing the need for further studies to assess its efficacy in broader patient populations [119].

##### AB-729

AB-729 is a triantennary N-Acetylgalactosamine (GalNAc)-conjugated siRNA targeting HBV transcripts that has been shown to suppress all HBV RNA transcripts, including those encoding HBsAg, HBx, and the HBV polymerase, resulting in a reduction in HBV antigens and replication [124]. In a phase 2 clinical trial, non-cirrhotic patients received varying doses of AB-729 every 4, 8, or 12 weeks in addition to NA therapy. HBsAg levels declined across dosing regimens, with 17 out of 31 participants achieving HBsAg levels below 100 IU/mL for at least 20 weeks after the last AB-729 dose. However, baseline HBsAg levels in the enrolled population were relatively low, with mean levels ranging from 3.14 to 3.37 log_10_ IU/mL (approximately 1380 to 2344 IU/mL) across cohorts. This may have influenced treatment outcomes and limits the generalizability of the results, particularly in patients with higher baseline HBsAg levels. Neither HBeAg status nor DNA positivity at baseline was associated with the treatment response. Although one participant achieved HBsAg seroconversion following AB-729 treatment, this isolated event occurred in a patient with low baseline HBsAg levels, highlighting the need for further studies to clarify its potential for sustained antiviral effects, particularly in populations with higher baseline HBsAg levels [125].

Beyond enhancing hepatic delivery, GalNAc conjugation may indirectly influence immune activation in a platform-dependent manner. Oligonucleotides such as antisense oligonucleotides (ASOs) and small interfering RNAs (siRNAs) can stimulate pattern recognition receptors (PRRs), including toll-like receptors (TLRs), leading to innate immune activation via TLR3, TLR7, or TLR9 [126]. While some have proposed that GalNAc may act as an adjuvant, there is no published evidence directly linking GalNAc conjugation to enhanced PRR signaling. Instead, GalNAc modifies biodistribution: a total of ~70% of unconjugated ASOs accumulate in immunoreactive cells and ~30% in hepatocytes, whereas GalNAc-conjugated ASOs show the reverse pattern, leading to reduced immune stimulation. This may explain why bepirovirsen, which remains active only in its unconjugated form, likely exerts its effect through immune activation rather than antisense activity alone. Supporting this, a GalNAc-conjugated ASO failed to show efficacy in HBV despite favorable pharmacokinetics [127]. In contrast, GalNAc-siRNA conjugation increases exposure to immune-responsive liver cells, such as Kupffer cells and liver sinusoidal endothelial cells, potentially enhancing innate immune responses via TLR3. Further research is needed to determine whether these effects are truly immunomodulatory or primarily due to pharmacokinetic shifts. Preliminary data from a phase 2a trial of 43 HBeAg patients treated with AB-729 combined with PEG-IFNα were presented in an abstract form. There was a mean HBsAg decline of 1.8 log_10_ at week 48 of treatment, with 80.5% of patients reaching HBsAg levels below 100 IU/mL, and two patients had HBsAg levels below the LLOQ [128]. Similar results were observed in the MARCH Part B trial with VIR-2218 + PEG-IFNα, where 15% of participants achieved HBsAg loss at the end of treatment (EOT), and post-treatment data suggested sustained declines in HBsAg, with a subset achieving functional cure endpoints. This comparison highlights the potential for siRNA-based therapies in combination with PEG-IFNα to achieve clinically relevant HBsAg reductions and stimulate HBV-specific immunity, supporting their role in functional cure strategies for CHB [119].

##### Nucleic Acid Polymers

Nucleic acid polymers (NAPs) block the assembly of subviral particles from hepatocytes and inhibit HBsAg replenishment in the blood [129,130]. A phase 2 study assessed the safety and efficacy of the NAPs REP 2139 or REP 2165 in combination with TDF and PEG-IFNα in HBeAg-negative CHB patients. Initially, all participants underwent 24 weeks of TDF monotherapy, followed by either 48 weeks of experimental therapy with TDF, PEG-IFNα, and NAPs REP 2139 or REP 2165, or 24 weeks of control therapy with TDF and PEG-IFNα and then 48 weeks of experimental therapy. A functional cure, defined as HBsAg below 0.05 IU/mL, HBV DNA not detected, and normal ALT levels, was achieved in 35% (14/40) of participants 48 weeks post-treatment cessation [47]. Additionally, these patients had no detectable HBV RNA or HBcrAg [35]. A viral kinetics study identified three patterns of HBsAg decline under NAPs including monophasic, biphasic and non-responders. All patients who had a functional cure had a rapid monophasic decline in HBsAg, suggesting that on-treatment viral kinetics might serve as a guide to NAP treatment [48].

##### VIR-3434

The monoclonal antibody VIR-3434 targets HBsAg and was found to effectively neutralize HBV and HDV in vitro. In experiments with human liver chimeric mice, VIR-3434 inhibited HBV dissemination during the spreading phase of infection and reduced circulating HBsAg and HDV RNA during the chronic phase [131]. In a phase 2 trial that combined VIR-3434 with VIR-2218, a hepatocyte-targeted siRNA [119] with or without PEG-IFNα achieved HBsAg loss in up to 15% of participants at 24 weeks. Additionally, anti-HB titers increased in many of the participants receiving the triple combination therapy, suggesting that VIR-3434 stimulates HBV-specific immune responses [119].

#### 3.5.2. Replication Inhibition

##### Capsid Assembly Modulators (CAMs)

CAMs target HBV replication by interfering with nucleocapsid formation, with two primary mechanisms: Class 1 CAMs accelerate normal capsid assembly but produce empty capsids devoid of a viral genome, while Class 2 CAMs induce the formation of structurally defective capsids that prevent proper viral replication and cccDNA recycling [132]. Linvencorvir is a novel CAM that was assessed in combination with NA (ETV, TAF, or TDF) and PEG-IFNα therapies in a multicenter phase 2 study [133]. The study included 72 patients with CHB across three cohorts: Cohort A included NA-suppressed patients who received NA and linvencorvir (n = 32); Cohort B comprised treatment-naïve patients who received NA and linvencorvir without PEG-IFNα (n = 10), and Cohort C involved treatment-naïve patients who received NA, linvencorvir, and PEG-IFNα (n = 30). Linvencorvir was dosed at 600 mg daily for 48 weeks, followed by 24 weeks of NA monotherapy or without NA if stopping criteria were met. No patients achieved the primary endpoint of HBV DNA < LLOQ with HBsAg loss at 24 weeks post-treatment. However, HBV DNA levels were suppressed below the LLOQ in all patients in Cohort B and 24/28 (86%) of patients in Cohort C by week 48. HBV RNA was also suppressed below the LLOQ in 14/15 (92%) of patients with quantifiable baseline HBV RNA by week 48, with sustained RNA responses observed during the follow-up in treatment-naïve patients. Rebounds in HBV RNA following treatment cessation were observed across all cohorts. The data indicate that while linvencorvir effectively suppressed HBV DNA and RNA in the study, it did not achieve a functional cure in combination with NA or PEG-IFNα [133].

#### 3.5.3. Entry Inhibitors

##### Bulevirtide

Bulevirtide (BLV) is a synthetic myristoylated peptide derived from the pre-S1 domain of the HBV large surface protein. BLV specifically binds the sodium taurocholate co-transporter polypeptide (NTCP) receptor and blocks HBV and hepatitis D virus (HDV) entry [134,135,136,137]. BLV may also have a secondary effect of reducing HBV and HDV clearance by the liver [138]. While BLV was approved for the treatment of compensated chronic HDV infection in Europe in 2020, its clinical use in combination with NA and/or PEG-IFN therapy in HBV mono-infected patients is yet to be explored. One potential use of BLV could be in patients undergoing liver transplantation (LT) due to HBV-related liver diseases, which could potentially help to prevent the infection of the liver graft [139]. As an entry inhibitor, BLV adds a novel mechanism to the HBV treatment armamentarium, complementing existing therapies such as nucleos(t)ide analogs and interferon-based regimens.

##### HBIG

The use of hepatitis B immunoglobulin (HBIG) and NA has drastically reduced HBV recurrence after LT, leading to patient and graft survival rates comparable to other indications for LT [140].

HBIG is a purified human plasma-derived IgG preparation rich in anti-HB antibodies. Its exact mechanism is not fully defined, but HBIG is thought to neutralize circulating HBV to prevent viral entry by binding to hepatocyte NTCP receptors and possibly eliminate infected cells via antibody-mediated immune mechanisms [141]. Additional proposed mechanisms include complement activation following HBsAg–anti-HB binding, enhanced humoral immunity, and the direct suppression of HBV replication [142].

When HBIG is combined with potent NAs such as entecavir or tenofovir disoproxil fumarate, HBV recurrence rates fall below 5%, even in patients with detectable viremia at the time of transplant [143]. Additionally, various studies on the synergistic effect of HBIG and NA prophylaxis for CHB recurrent have found general recurrence rates to be below 10%, which is lower than the rates seen in HBIG or NA monotherapy. As a result, combined prophylaxis involving combinations such as HBIG and NA has become the standard of care in patients who have undergone LT [143]. Like BLV, HBIG also contributes to HBV entry inhibition, further reinforcing the importance of targeting viral entry in HBV management. Further information about HBV entry inhibitors, including the pharmacologic and molecular mechanisms of entry inhibitors, especially those which target NTCP-mediated viral infection, can be found in the review by Li et al. [144].

In summary, currently approved therapies, including PEG-IFNα and NA, are associated with low rates of HBsAg loss and functional cures. New treatments with different mechanisms of action are in development. Several of these emerging therapies have shown promising preliminary results, and combing agents from different classes might improve their efficacy. The treatments discussed in this section are summarized in Table 4.

**Table 4:** Summary of treatments and corresponding functional cure rates.

#	Treatment Type	Sample Size and Patient Characteristics	% Functional Cure	Key Findings from the Studies	Patient Response	Refs.
1	PEG-IFNα monotherapy added to existing NA therapy	N = 185 (HBeAg+ with undetectable HBV DNA on an NA regimen of >1 year)	7.8% at 96w	PEG-IFNα tx did not significantly augment HBsAg clearance.	HBsAg loss: 7.87% (PEG-IFN, 7/90) vs. 3.2% (NA alone, 3/93); all had undetectable HBV DNA with PEG-IFN.	[93]
2	Switching from ETV to PEG-IFNα	N = 200 (ETV for 9–36 months, undetectable HBV DNA, and HBeAg+)	8.5% at 48w	Better outcomes in the HBsAg < 200 IU/mL group.	HBsAg seroconversion: 4.3% (4/94) at week 48; HBV DNA < 1000 copies/mL in 72.0%.	[94]
3	NA monotherapy (LAM, ETV, TDF, and TAF)	ETV: N = 658 (36% HBeAg+); TAF vs. TDF: N = 875 (HBeAg+); TAF vs. TDF: N = 426 (HBeAg−); ETV: N = 146 (HBeAg+)	1.4–8%	Long-term treatment with NA required for results.	TDF: 8% HBsAg loss over 3 years (HBeAg+).	[129,130,131,132]
					ETV: HBsAg loss over 5 years (HBeAg+) and 4.6% over 5 years (HBeAg−).	
					TAF: a 1% HBsAg loss over 5 years (HBeAg+).	
4	NA discontinuation after 4–5y of tx	N = 42 (HBeAg−, TDF); N = 691 (HBeAg−, various NAs); Meta-analysis: N = 1753 (HBeAg−, various NAs across 17 studies)	13–19%	HBsAg loss observed post-therapy.	In HBeAg-negative patients, HBsAg loss after NA discontinuation (4–5 years) varied: 39% (adefovir, Hadziyannis et al.), 19% (TDF, Berg et al.), 13% (6-year rate, Papatheodoridis et al.), and 33.1% (Chen et al.).	[101,103,105,106]
5	Bepirovirsen	N = 457 (NA therapy or naïve)	9–10%	The 24-week treatment with bepirovirsen led to sustained HBsAg and HBV DNA loss in 9–10% of participants with CHB.	A total of 9–10% of patients had sustained loss of HBsAg; by the end of treatment, this was seen in 59% to 63% of participants receiving 300 mg.	[110]
6	Si-RNA JNJ-3989 and capsid modulator JNJ-6379	N = 130 (HBeAg−, non-cirrhotic); N = 470 (non-cirrhotic, virologically suppressed, HBeAg+/−); N = 84 (treatment-naïve or NA-suppressed, HBeAg+/−)	0%	The dose-dependent response rarely resulted in complete HBsAg loss.	Tx with JNJ-3989 led to HBsAg reductions of ≥1 log_10_ IU/mL in a high percentage of patients, sustained in 38%.	[111,112,133]
7	AB-729 ± NA ± PEG-IFNα	N = 16 (CHB, on NA therapy); N = 32 (CHB, HBeAg+, treated with Ab-729, sustained HBsAg suppression); N = 43 (virally suppressed, HBeAg−)	0%	Sustained declines in HBsAg levels with some patients showing temporary seroconversion.	A total of 93% had HBsAg < 100 IU/mL, while 9.7% had HBsAg below the LLOQ but no sustained loss.	[120,121,122,133]
8	VIR-2218 and PEG-IFNα	N = 18 (CHB, HBeAg status unknown)	16%	A significant increase in HBsAg loss with combination therapy.	A total of 30% achieved HBsAg loss, and 16% sustained loss	[115]
9	NAPs (REP 2139, REP 2165) ± TDF, PEG-IFNα	N = 40 (non-cirrhotic, HBeAg−)	35%	High rates of a functional cure in the REP 401 phase II study. A non-monophasic HBsAg decline pattern had a 100% negative predictive value for the functional cure.	Sustained functional cure observed in 14/40 (35%) patients.	[43,123,124]
10	VIR-3434 + VIR-2218 ± PEG-IFNα	N = 21 (HBeAg status unknown)	15%	VIR-3434 combined with VIR-2218 and optionally PEG-IFNα increased HBsAg loss rates.	Up to 15% showed HBsAg loss at 24w.	[115]
11	Linvercorvir (CAM) ± NA ± PEG-IFNα	N = 72; Cohort A: NA-suppressed (N = 32); Cohort B: Treatment-naïve, NA + linvencorvir (N = 10); Cohort C: Treatment-naïve, NA + linvencorvir + PEG-IFNα (N = 30)	N/A	- Linvencorvir dosed at 600 mg daily for 48 weeks.	HBV DNA was suppressed, but no evidence of a functional cure.	[127]
				- HBV DNA < LLOQ: All patients in Cohort B and 86% (24/28) in Cohort C by week 48.		
				- HBV RNA < LLOQ: a total of 92% (14/15) of patients with quantifiable baseline HBV RNA by week 48.		
				- No HBsAg loss at 24 weeks post-treatment.		
				- HBV RNA rebound observed across all cohorts post-treatment cessation.		

A functional cure was defined as sustained HBsAg loss along with HBV DNA levels remaining below the LLOQ for at least 6 months post-treatment.

## 4. Studies and Publications That Have Examined the Use of Vaccines in Attempting to Achieve a Functional Cure

The utilization of vaccines has emerged as a potential approach in the quest to achieve a functional cure for HBV. The first prophylactic vaccines against HBV were plasma-derived vaccines that were developed in France and in the USA in the early 1980s using HBsAg particles harvested from the blood of carriers of HBsAg and then purified [145]. However, concerns involving the high production cost of the vaccine coupled with concerns that the plasma used to derive HBsAg may be contaminated with other diseases such as HIV led to the development of the recombinant HBV vaccine and its subsequent approval in 1986 by the FDA [146]. Traditionally, these prophylactic vaccines have been used to prevent the initial infection with HBV. However, this section will discuss therapeutic vaccinations for HBV which differ in their aim. Therapeutic vaccines are administered to patients with existing CHB with the intention of achieving a functional cure. These types of vaccines deliver the HBV antigen in a non-infectious form to initiate or improve existing adaptive immune responses that are targeted to HBV.

### Therapeutic Vaccines

Efforts to develop a therapeutic vaccine for HBV began with the use of recombinant vaccines. These vaccines contained the pre-S2 and S HBV proteins [147] that can elicit a robust immune response, particularly the generation of HBsAb and the stimulation of CD4^+^ T cells [148]. Studies on HBV vaccines for the treatment of HBV showed reductions in HBV levels but did not achieve a functional cure [149,150,151,152]. Subsequent studies combining HBsAg vaccines with hepatitis B immunoglobulin were unsuccessful in reaching a functional cure [153,154,155,156,157]. As the pursuit of a functional cure through recombinant protein-based HBV vaccines has proven largely unsuccessful, the focus has shifted towards exploring other novel vaccination strategies.

Several therapeutic vaccine strategies have been studied, each demonstrating limited efficacy in clinical trials. DNA vaccines, which incorporate genes encoding HBV antigens into a plasmid DNA expression vector, initially showed promise by stimulating antigen-presenting cells and facilitating the activation of B cells, CD4+ T cells, and CD8+ T cells [158]. Despite early success in animal models, these vaccines did not achieve similar results in human trials [158,159,160,161,162,163,164,165], with further studies of modified versions like HB-100 and HB-110 also failing to deliver a functional cure [166,167].

Another approach involved yeast-based vaccines, specifically GS-4774, which contained the HBV antigens S, X, and C. This vaccine did not induce a functional cure and elicited little to no HBsAg response, even when used in combination with nucleoside analogs or the PD-1 inhibitor, nivolumab [75,168,169]. Similarly, adenovirus vaccines such as TG1050, which carries a fusion protein of the HBV core and envelope domains, yielded promising results in murine models by inducing long-lasting HBV-specific T cells. However, these findings could not be replicated in human trials, where only minor decreases in HBsAg were observed with no significant suppression of HBV DNA [170].

The development of lipopeptide epitope-based vaccines, like CY-1899, targeted at cytotoxic T lymphocytes also failed to make a significant impact [put 155, 156 here]. While CY-1899 induced specific CD8+ T cells in healthy subjects, it did not reduce HBV DNA levels or result in the loss of HBsAg in CHB patients [171,172] [this should be 156 alone]. Lastly, vaccines using autologous dendritic cells showed some promise in reducing HBV DNA levels in a portion of CHB patients, but these results were not universally replicable across all patient groups, highlighting the challenges in achieving a consistent functional cure [173].

VBI-2601 (BRII-179) is a therapeutic vaccine comprising recombinant pre-S1, pre-S2, and S HBV antigens designed to elicit both B-cell and T-cell responses [34]. VBI-2601 was evaluated in a phase 2 study for CHB treatment in combination with GalNAc-siRNA BRII-835 (VIR-2218), which targets viral RNA to reduce protein expression and alleviate immune suppression. The combination induced anti-HB titers in ≥40% of participants, with titers exceeding 100 IU/L by week 40, and elicited robust T-cell responses in up to 70% of patients. However, HBsAg reductions (~1.77–1.81 log_10_) were insufficient to achieve a functional cure, with only two patients achieving HBsAg seroclearance. Despite its favorable safety profile, the data regarding VBI-2601 highlight the need for further refinement to enhance durable virologic control and treatment outcomes [34].

TherVacB is a therapeutic vaccine that utilizes a heterologous protein-prime and Modified Vaccinia Ankara (MVA)-vector boost approach. This strategy incorporates HBsAg and HBcAg particulates to stimulate robust and targeted immune responses against HBV. Preclinical studies in HBV-infected murine models demonstrated the vaccine’s capacity to elicit strong B-cell and T-cell responses, effectively breaking immune tolerance. The inclusion of amino acid-based formulations has further enhanced vaccine stability, ensuring the preservation of immunogenicity even under thermal stress, a critical factor for global distribution. While TherVacB achieved anti-HB seroconversion and robust activation of HBV-specific CD8+ T cells, viral clearance remained incomplete, highlighting the ongoing need for optimization to achieve sustained virologic control and a functional cure [174,175].

To enhance the efficacy of TherVacB, researchers have explored combining it with immune checkpoint inhibition to restore exhausted HBV-specific T-cell responses. A study from Bunse et al. investigated the effect of PD-L1 silencing using siRNA in combination with TherVacB in a murine model of chronic HBV infection [176]. Their findings demonstrated that PD-L1 downregulation significantly increased the functionality of HBV-specific CD8+ T cells, leading to enhanced antiviral immune responses and a greater control of HBV replication in both peripheral blood and liver tissue. Notably, PD-L1 silencing during the priming phase of vaccination resulted in a more pronounced antiviral effect than inhibition during the boost phase, underscoring the importance of early immune modulation in therapeutic vaccination strategies. These results suggest that immune checkpoint blockades may serve as a critical adjunct to therapeutic vaccination by improving T-cell responses [176].

In summary, to date therapeutic vaccines have not resulted in clinically significant rates of functional cure. Nonetheless, efforts are still underway to refine existing therapeutic vaccine strategies and to explore novel agents. Ongoing clinical trials of therapeutic vaccines are listed in Table 5 below.

**Table 5:** Summary of therapeutic vaccines currently in clinical trials.

Item #	Vaccine Name	Description	Trial Phase	Citation
1	GSK3528869A	Viral vector vaccine in patients on NA.	1/2	[177]
2	GSK3228836	Sequential treatment with GSK3228836, an antisense oligonucleotide, and the viral vector vaccine GSK3528869A in participants on NA.	2	[178]
3	ChAdOx1-HBV and MVA-HBV (VTP-300)	Chimpanzee adenovirus-vectored vaccines (ChAdOx1)-HBV and Modified Vaccinia Ankara (MVA)-HBV vaccines with or without nivolumab in patients with viral suppression.	1b/2a	[179]
4	HepTcell (FP-02.2)	The HepTcell (Adjuvanted FP-02.2) vaccine in treatment-naive patients with HBeAg-negative CHB in the low replicative state with low HBsAg levels (qHBsAg ≥ 10 IU/mL but ≤100 IU/mL in the 12 months prior to screening). Double-blind, randomized, placebo-controlled design.	2	[180]
5	VBI-2601 (BRII-179)	A therapeutic vaccine containing recombinant pre-S1, pre-S2, and, S HBV antigens, evaluated in combination with GalNAc-siRNA BRII-835 (VIR-2218) to enhance immune responses. Induced anti-HBs in ≥40% of patients and T-cell responses in up to 70% but achieved modest HBsAg reductions (~1.8 log_10_) with limited seroclearance (two patients).	2	[34]

Patients with low HBsAg titers.

Population surveys have demonstrated that the average circulating HBsAg in patients with chronic HBV infection is ~10,000 IU/mL, with patients having low baseline HBsAg of <1000 IU/mL representing 5% of the patient population [181,182,183,184,185,186,187,188]. These low-prevalence patients are qualitatively different from the larger population of HBV patients because of the role circulating HBsAg levels play in suppressing immune function. Functional cure rates as high as 30% were reported in patients with low HBsAg titers treated with pegIFN [120,121,122,123]. It is not known whether results of clinical trials that select for patients with low HBsAg titers will be generalizable to the larger population of HBV patients.

## 5. Conclusions

Functional cure can be defined as undetectable HBsAg and HBV DNA with normal ALT persisting at least six months after the completion of therapy [22]. Currently available treatments including PEG-IFNα and NA achieve low rates of a functional cure. There are some interesting data regarding a functional cure after the discontinuation of long-term NA therapy, particularly in patients who develop a clinical relapse. New therapeutic agents are in development with diverse mechanisms of action including oligonucleotides, siRNA, NAPs, monoclonal antibodies, capsid assembly modulators, therapeutic vaccines, and immune checkpoint inhibitors. The complexity of the HBV lifecycle and immune evasion strategies necessitate a multifaceted approach, integrating novel therapies with existing treatments.

While therapeutic advances are critical, expanding HBV screening and vaccination efforts remains essential to reducing the disease burden. Universal once-in-a-lifetime HBV screening has been proposed as a cost-effective strategy to identify undiagnosed infections and guide targeted interventions [189]. Additionally, broader implementation of timely birth-dose vaccination could further reduce new infections, particularly in high-prevalence regions [189].

The global impact of HBV, particularly in regions with high prevalence, highlights the urgency of these efforts. Future research should focus not only on developing more effective treatments but also on understanding the pathogenesis and viral–host interactions of HBV to provide the basis to tailor therapies more precisely. A comprehensive approach will be crucial in transforming the management of CHB and moving closer to the ultimate goal of eradicating HBV.

## Copyright Statement

D.G.S. is a military service member. This work was prepared as part of my official duties. Title 17 U.S.C. 105 provides that “Copyright protection under this title is not available for any work of the United States Government”. Title 17 U.S.C. 101 defines a United States Government work as a work prepared by a military service member or employee of the United States Government as part of that person’s official duties.
